# Experienced Weight Stigma Changes Following Bariatric Surgery: a Systematic Review and Meta‐Analysis

**DOI:** 10.1111/obr.70179

**Published:** 2026-06-16

**Authors:** Hannah R. Farnsworth, Dakota L. Leget, Lara J. LaCaille, Rick A. LaCaille, Afton M. Koball, Alicia Niemann, Chloë Z. Backderf, Carly R. Mastrian, Ellie M. Von Duyke

**Affiliations:** ^1^ Department of Physical Medicine and Rehabilitation University of Utah Health Salt Lake City Utah USA; ^2^ Department of Clinical and Health Psychology University of Florida Gainesville Florida USA; ^3^ Department of Psychology University of Minnesota Duluth Duluth Minnesota USA; ^4^ Department of Psychiatry and Psychology Mayo Clinic Rochester Minnesota USA

**Keywords:** meta‐analysis, metabolic bariatric surgery, weight bias, weight loss, weight stigma

## Abstract

**Background:**

Experiencing weight stigmatization is linked to maladaptive eating, poorer mental health, and increased risk of weight gain, potentially impacting metabolic bariatric surgery (MBS) outcomes. This meta‐analysis aimed to synthesize patient experiences of weight stigmatization before and after MBS and to examine the association of presurgical weight stigma on postsurgical weight loss.

**Methods:**

This preregistered study followed PRISMA guidelines. Six databases were searched through March 2025; eligible studies included published articles and dissertations/theses available in English that quantitatively measured presurgical weight stigma among MBS samples and included relevant outcomes. Meta‐analyses utilized random effects models with change scores converted to Hedge's *g* and associations between presurgical experienced weight stigma and post‐MBS weight change converted to coefficient *r*.

**Results:**

Forty‐one studies were included, with 36 reporting on experienced weight stigma change pre‐ to post‐MBS and 10 studies reporting on the relationship between pre‐MBS experienced weight stigma and post‐MBS weight loss.

**Conclusions:**

Results revealed weight stigmatization decreased substantially after MBS (6–12 months, *g* = 1.28; 13–24 months, *g* = 1.16; 25–36 months, *g* = 1.26). Presurgical weight stigma was not associated with weight loss post‐surgery (*r* = 0.05). Findings are limited by minimal studies reporting on longer term relationships between baseline stigma, post‐MBS weight loss, and additional health outcomes.

## 1 Introduction

Weight stigma occurs when individuals are mistreated or discriminated against because of their body shape or weight. Approximately half of adults report experiencing weight stigma in their lifetime [1], with people in larger bodies reporting the highest rates of weight‐based teasing and maltreatment [2]. Similar to other forms of social stigma, weight stigma is considered a psychophysically stressful experience, with prior research demonstrating increases in negative affect [3], acute inflammation biomarkers [4], and salivary cortisol levels [3, 5] following exposures to weight stigma. Experiences of weight stigma can activate stress pathways and prompt metabolic, emotional, and behavioral changes that increase vulnerability to developing adverse health and well‐being outcomes [6, 7]. Additionally, individuals who have experienced weight stigmatization may also delay recommended healthcare screenings, postpone or avoid necessary care, or switch providers frequently due to felt stigma and fears of encountering future stigmatization [7, 8, 9]. Prior research suggests that weight stigma increases one's risk of developing obesity and obesity‐related morbidities and mortality, highlighting the potential deleterious effects [6]. These findings raise concerns about the impact of weight stigma among individuals pre‐ and post‐metabolic bariatric surgery (MBS) given the higher weight status of patients seeking MBS.

MBS is considered the most effective intervention for reducing weight and related comorbidities among persons with obesity (BMI ≥ 35 kg/m^2^ or BMI of 30–34.9 kg/m^2^ with comorbidities) [10]. For many, MBS results in improvements in physical health conditions, such as type 2 diabetes, hypertension, dyslipidemia, and musculoskeletal pain, in addition to long‐term weight loss and improved functional mobility [11, 12]. Common reasons patients seek MBS include physical complaints, mobility impairments, health concerns, challenges engaging in daily activities (e.g., walking, playing with grandchildren), and improve body image concerns [13, 14, 15]. Another reason for considering MBS is the goal of minimizing experiences of weight‐related stigma [13, 14, 15]. Interestingly, despite the well‐documented health benefits of MBS, individuals report ongoing and uniquely challenging stigmatizing experiences following surgery, including beliefs from others that having MBS is the “easy way out” [16]. For those who continue to experience weight stigma, current evidence suggests that health benefits of MBS could be compromised by weight stigmatization given that experiencing weight‐based mistreatment is associated with greater disordered eating behavior (e.g., night eating, binge eating), poorer body image, and higher depression and anxiety levels among individuals who have had MBS [17].

A systematic review by Bennett and colleagues [17] summarized studies that examined weight stigma among patients undergoing MBS; most studies were cross‐sectional (pre‐ *or* post‐MBS) with only two longitudinal studies included, revealing a significant gap in the literature to date. Their review found that experienced and internalized weight stigma was associated with psychological, behavioral, and physical health problems among individuals pursuing or having undergone MBS [17]. The present study expands on the work of Bennett and colleagues [17] by updating and expanding the literature review, analyzing the change in patient experiences of weight stigmatization before and after MBS using data from a larger pool of longitudinal studies than was previously reported, and by investigating the predictive impact of presurgical weight stigmatization on postsurgical outcomes. Based on the results reported in Bennett and colleagues [17], we hypothesized that weight‐based stigma would improve from pre‐to‐post MBS. To expand on previous results, we planned to examine moderators including demographic variables and surgery type. We also hypothesized that greater weight stigmatization pre‐MBS would predict poorer outcomes post‐MBS.

## 2 Methods

### 2.1 Search Strategy and Selection Criteria

This meta‐analysis followed the guidelines set forth by the Preferred Reporting Items for Systematic Reviews and Meta‐Analyses (PRISMA) [18]. The review protocol was registered through Open Science Framework (OSF; https://osf.io/q7hmc/). Databases searched included CINAHL, Google Scholar, ProQuest Dissertations and Theses Global, PsycINFO, PubMed, and the Web of Science Core Collection. Two systematic searches were conducted using a combination of (1) an abstract and title search using related terms and synonyms for weight stigma and bariatric surgery and (2) a full‐text search of specific weight stigma measures alongside the search terms “bariatric surgery” or synonyms (see Table S1 for details about search terms and search strategy). Databases were initially searched from December 16, 2022, to March 13, 2023, fully updated on March 23, 2025, with supplemental searches (i.e., search alerts, forwards, and backwards citation searching) occurring simultaneously. Data are available upon request from the corresponding author.

**FIGURE 1:**
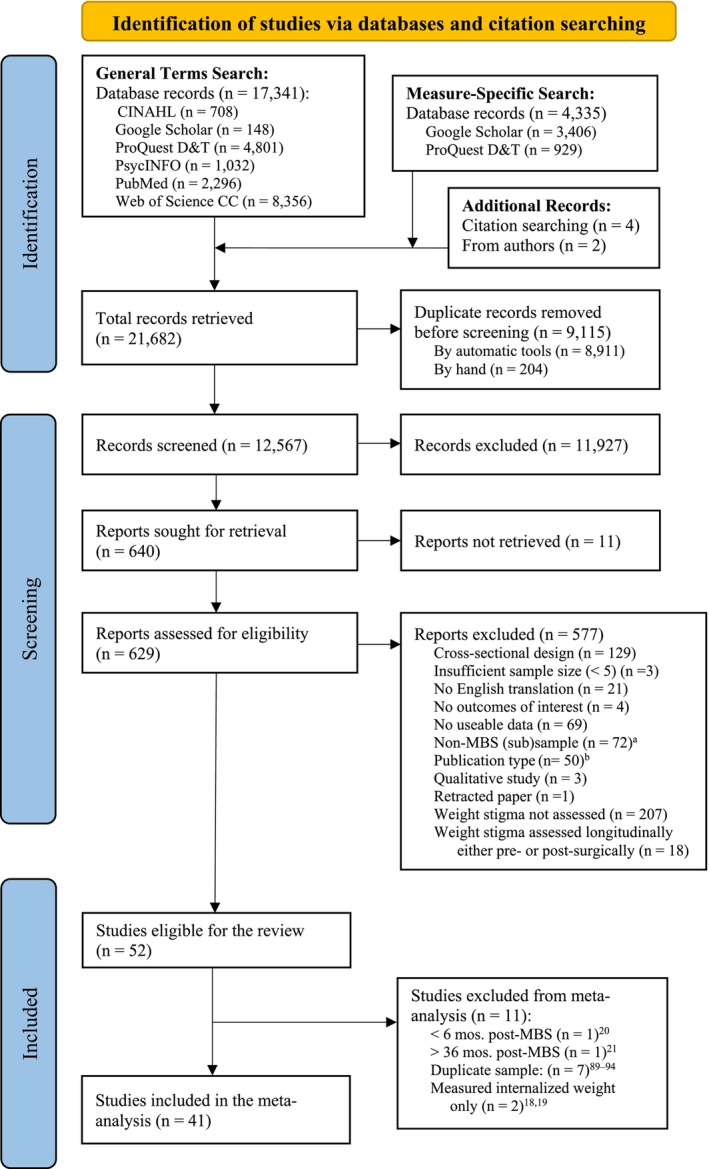
PRISMA flow chart. ^a^Nonsurgical samples and temporary MBS procedures (e.g., gastric balloon). ^b^Publication type included anecdotal/opinion papers, best practice reports, conference abstracts, reviews, and protocol papers.

This meta‐analysis included studies using single‐group pre–post, quasi‐experimental, and randomized controlled trials. Published articles and dissertations or theses were eligible for inclusion provided they (a) reported on an MBS sample, (b) included a measure of weight stigma captured pre‐MBS, (c) reported on an outcome of interest (i.e., postsurgical weight stigma, weight loss), and (d) there was an English translation available. Details on the number of studies excluded and included are outlined in the PRISMA flow diagram in Figure 1. Studies were excluded if they reported exclusively on qualitative data, used a cross‐sectional or post‐test‐only design, measured presurgical weight stigma retrospectively, did not offer a full‐text English translation, did not include an MBS sample, did not measure weight stigma and any outcome of interest, or if data were reported on less than 10 participants (i.e., case studies), or did not meet the previously stated inclusion criteria. Samples in which nonsurgical MBS procedures (e.g., gastric balloon, endoscopic sleeve gastroplasty, implanted devices, bariatric embolization of arteries for the treatment of obesity [BEAT], implanted devices) were used were not included. Regarding outcomes, we initially took a broad approach, including studies that measured any outcome (e.g., mental health, eating behavior, quality of life, medical outcomes; assuming they met all other inclusion criteria); however, the only outcome that was reported on by more than two studies was post‐MBS weight loss. Thus, studies that only reported on other outcomes were eventually excluded from the meta‐analysis. We also excluded studies that only measured internalized weight stigma, as only two studies measured this variable pre‐ and post‐MBS [19, 20]. Similarly, we chose to exclude studies that only reported on outcomes < 6 months [21] or > 3 years [22] post‐surgery, due to insufficient study numbers.

Authors were contacted (up to three times) if they indicated collecting but did not report on longitudinal data and public distress scale scores that could be included in the prediction component of the meta‐analysis. Of the 46 authors contacted, 31 did not reply, eight replied but were unable to provide the data, one sent a manuscript published after the literature search was conducted with pre‐post data [19], four sent the requested analysis [20, 21, 22, 23], one provided the dataset to run the analyses ourselves [24], and for the TeenLABS study [25], we were granted access to the database [26] through the NIDDK Central Repository, which allowed for further analyses. Five members of the research team were involved in independently screening the abstracts, with two researchers screening every abstract. Disagreements were settled through discussion and consultation with other team members. A total of 41 studies were included in the review, with 36 reporting on change in weight stigma perceptions from pre‐ to post‐MBS. Of these, 26 studies (number of participants, *N* = 2833) reported on changes that occurred 6 to 12 months after surgery (hereafter referred to as T2), 16 studies (*N* = 3480) reported on changes over 13 to 24 months (T3), and eight studies (*N* = 2016) reported on changes over 25 to 36 months (T4). Ten studies (*N* = 2737) provided data examining the relationship between pre‐MBS weight stigma and post‐MBS weight change (three of which also provided pre–post changes in weight stigma).

### 2.2 Data Extraction

Coding variables included year, design, country, sample size, type of surgery, demographics (age, gender, and race), baseline BMI, weight stigma measure, post‐MBS outcomes, and time of follow‐up. Quantitative data captured from the pre–post studies included any data that could be used to estimate hedges *g* (means and standard deviations, change scores, inferential statistics [e.g., *t*, *f*]). For the analyses examining pre‐MBS weight stigma predicting post‐surgery outcomes, we recorded Pearson *r* coefficients.

### 2.3 Outcome Measures

All studies included in the meta‐analysis used the Impact of Weight on Quality of Life‐Lite (IWQoL‐Lite) [27] to measure stigma. In the adult version, the 5‐item Public Distress (IWQoL‐PD) subscale was used to capture experiences of perceived stigma. Questions asked about perceived personal and societal discrimination, such as worrying about fitting through turnstiles or aisles and experiencing ridicule or teasing due to one's weight [27]. This subscale is the most widely used measure of experienced weight stigma for patients undergoing MBS [17] and has demonstrated good reliability and validity [28]. For the three studies exploring adolescent populations, the 6‐item Social Life subscale of the youth version of this measure (IWQoL‐Kids) [29] was used. This subscale is similar to the IWQoL‐Lite for adults and includes questions asking about teasing from others due to weight [29].

For the studies that examined the association between pre‐MBS weight stigma and post‐MBS weight loss, we combined effect sizes of different measures of weight loss, including change in total body weight, BMI, or excess body weight.

### 2.4 Assessment of Study Quality

The quality of the studies was rated independently by two study coders, both of whom followed the guidelines presented in NHLBI Quality Assessment Tool for Before‐After (Pre–Post) Studies with No Control Group [30]. This tool was slightly modified to assess bias in intervention studies across seven items (see Table S2 for more information). Based on the standards specified for each of the seven items, the reviewers reported whether the study met each criterion, with one point allotted per criterion. If the research paper did not address a criterion, it received zero points for that category. Summing across the seven criteria provided an estimate of each study's overall quality, with higher scores indicating less risk of bias.

### 2.5 Meta‐Analytic Strategy

The data were analyzed using the Comprehensive Meta‐Analysis (CMA) version 4 software [31]. To assess the magnitude of change of scale scores (e.g., IWQoL‐PD), effect sizes were estimated by subtracting the postsurgical mean from the presurgical mean followed by dividing by the pooled and weighted standard deviation. More specifically, effect sizes were expressed as Hedge's *g* along with the 95% confidence interval. In cases where individual studies did not provide these means and standard deviations, effect size estimation was based on conversion formulas within the CMA software. Effect size calculation was adjusted such that a positive effect size indicates a better outcome. Because differences in sample and intervention characteristics may yield different effect sizes between studies, a random effects model was used. None of the studies provided a correlation among outcomes over time, so a correlation of 0.50 for each study was used, as recommended by Borenstein and colleagues [31]. For synthesis of the studies examining the association between presurgical weight stigma and post‐MBS weight change, reported values were examined using the correlation coefficient *r* along with the 95% confidence interval. Forest plots were generated to reflect the magnitude of the respective effect sizes.

Cochrane's *Q*‐statistic and the *I* ^2^ index were used for examining heterogeneity. Substantial heterogeneity may suggest potential moderation of effects that warrant further analyses. A subgroup analysis within CMA was used to examine the potential categorical moderator of surgery type. The quality of the studies (i.e., risk of bias) and gender of surgical patients (% female) were examined as moderators using meta‐regression due to the continuous nature of the variables. Egger's test and trim‐and‐fill procedures were used to detect funnel plot asymmetry and correct for potential missing studies (i.e., publication bias).

## 3 Results

### 3.1 Description of Studies

In total, 41 studies, published between 2004 and 2025, met the inclusion criteria and were included for meta‐analysis. A more detailed summary of the studies is included in Table 1.

**TABLE 1:** Summary of included studies.

Study authors (year)	Data source	Country	Surgery type	BMI (*M*)	Age (*M*)	Female (%)	Race (%)	Stigma measure	*N* Follow‐up analysis [*N* at baseline]	Follow‐up timing (mos.) used in analyses [other timepoints not analyzed]
Boan et al. [32] (2004)^a^	Journal	United States	RYGB	52.9	41.2	85	79 W	IWQoL‐Lite (PD)	40 [40]^d^	6
Lauzon [33] (2007)^b^	Dissertation	United States	RYGB	54.8	40.5	74.9	73.2 W 20.3 B 6.1 H 0.4 A	IWQoL‐Lite (PD)	126 [231]	12 [6, 24, 60–84]
Kolotkin et al. [34] (2009)^a^	Journal	United States	RYGB	47.3	42.1	84.3	89.3 W	IWQoL‐Lite (PD)	308 [421]	24
Zeller et al. [35] (2009)^a^	Journal	United States	RYGB	63.5	16.4	64.5	80.6 W 16.1 B 3.2 O	IWQoL‐Kids (SL)	28–29 [31]	12 [6]
Osei‐Assibey et al. [36] (2010)^a^	Journal	United Kingdom	LAGB	NR	NR	NR	NR	IWQoL‐Lite (PD)	22 [27]	6+
Fezzi et al. [37] (2011)^a^	Journal	France	LSG	47.0	42.5	77.1	NR	IWQoL‐Lite (PD)	75 [78]	12
Therrien et al. [38] (2011)^a^	Journal	Canada	BPD/DS	52.6	45	79	NR	IWQoL‐Lite (PD)	48 [67]	12
Hsu [39] (2012)^a^	Dissertation	United States	LAGB 28% RYGB 72%	47.3	40.2	92	40.9 B 38.6 H 15.9 W 2.3 A 2.3 O	IWQoL‐Lite (PD)	88 [307]	12
Davis [40] (2013)^a^	Thesis	Australia	LAGB	41.1	47.5	80	NR	IWQoL‐Lite (PD)	40 [152]	21.1^c^; min. 12
Aftab et al. [41] (2014)^b^	Journal	United Kingdom	LAGB	52.6	50.3	84.6	NR	IWQoL‐Lite (PD)	26 [76]	12
Crowley [42] (2014)^b^	Dissertation	United States	RYGB	NR	47.3	91.7	68.8 W 29.2 B 2.1 H	IWQoL‐Lite (PD)	46 [75]	36
Miras et al. [43] (2015)^b^	Journal	United Kingdom	RYGB 80% VSG 7% LAGB 13%	48.5	46.5	81	NR	IWQoL‐Lite (PD)	90 [90]^d^	12
Chalut‐Carpentier et al. [44] (2015)^a^	Journal	Switzerland	RYGB	46.3	43	85	NR	IWQoL‐Lite (PD)	27 [38]	6
Courcoulas et al. [45] (2015)^b^	Journal and dataset	United States	RYGB 75% LAGB 25%	RYGB 46^c^ LAGB 44^c^	RYGB 46^c^ LAGB 48^c^	81.9	86.2 W 10.5 B 3.4 O	IWQoL‐Lite (PD)	2022 [2358]	36
Sarwer et al. [46] (2015)^a^	Journal	United States	RYGB	45.1^c^	48^c^	0 (all male)	100 W	IWQoL‐Lite (PD)	30 (12 M) 28 (24 M) 28 (36 M) [32]	12, 24, 36 [48]
Alsters [47] (2016)^a^	Dissertation	United Kingdom	VSG 32.6% RYGB 67.4%	46.8	46.5	72.2	65.9 W	IWQoL‐Lite (PD)	48 [169]	12 [3, 24]
Dixon et al. [48] (2016)^a^	Journal	United States	LAGB	35.6	40^c^	90.6	77.2 W 10.7 H 9.4 B 1.3 A 1.3 O	IWQoL‐Lite (PD)	140 (12 M) 132 (24 M) 126 (36 M) [149]	12, 24, 36 [48, 60]
Conason et al. [24] (2017)^a^ ^,^ ^b^	Journal & dataset	United States	RYGB 15.6% LAGB 84.4%	47.3	39.4	84.4	14 W 32 B 49 H 1 A 5 O	IWQoL‐Lite (PD)	84 (12 M) 57 (24 M) [327]	12, 24
da Silva et al. [49] (2017)^a^	Journal	Brazil	ESG	48.4	39.8	96	NR	IWQoL‐Lite (PD)	50 [50]^d^	12
Monpellier et al. [50] (2017)^a^	Journal	Netherlands	RYGB	44.5	45.8	82.5	NR	IWQoL‐Lite (PD)	1079 [2137]	24 [15]
Strain et al. [51] (2017)^a^	Journal	NR	BPD/DS	53.4	42.7	68.8	NR	IWQoL‐Lite (PD)	189 (12 M) 193 (36 M) [275]	12, 36 [60, 84, 108]
Wee et al. [52] (2017)^a^	Journal	United States	LAGB 46% RYGB 54%	46.8	44.4	76	71 W 17.5 B 11.4 H	IWQoL‐Lite (PD)	457 (12 M) 394 (24 M) [537]	12, 24
Zeller et al. [53] (2017)^a^	Journal	United States	RYGB	59.2	16.0	64.3	85.7 W	IWQoL‐Kids (SL)	14 [16]	24 [6, 12, 18, 72+]
De Raaff et al. [54] (2018)^a^	Journal	Netherlands	RYGB	44.7	43.5	87	NR	IWQoL‐Lite (PD)	276 [482]	15
Dixon et al. [55] (2018)^a^	Journal	United States and Canada	LAGB	45.4	44.4	79.3	NR	IWQoL‐Lite (PD)	592 (12 M) 464 (24 M) 404 (36 M) [652]	12, 24, 36 [48, 60]
Osman et al. [56] (2018)^a^	Journal	Saudi Arabia	LSG	46.8	33.2	45	NR	IWQoL‐Lite (PD)	100 [100]^d^	12
Preiss et al. [57] (2018)^a^	Journal	Australia	LAGB	42.6	42.6	80	NR	IWQoL‐Lite (PD)	88 [99]	6 [1, 2, 3, 4, 5]
Sarwer et al. [58] (2018)^a^	Journal	United States	LAGB 19.8% RYGB 80.2%	44.5^c^	41^c^	100	96.2 W 2.8 B 0.9 O	IWQoL‐Lite (PD)	80 (12 M) 60 (24 M) 87 (36 M) [106]	12, 24, 36 [48]
Geubbels et al. [59] (2019)^a^	Journal	Netherlands	RYGB	41.7	42.6	87	NR	IWQoL‐Lite (PD)	168 [220]	12
Robert et al. [60] (2019)^a^	Journal	France	RYGB 50% OAGB 50%	43.9	43.5	75	NR	IWQoL‐Lite (PD)	130 [234]	24
Alessimii [61] (2020)^a^	Dissertation	United Kingdom	RYGB	42.4	46.9	85.7	NR	IWQoL‐Lite (PD)	11 [14]	12 [3]
Moreno Gijón et al. [62] (2020)^a^	Journal	Spain	SG 15.7% RYGB 84.2%	46.5	45.7	67.1	NR	IWQoL‐Lite (PD)	57 [70]	12
Shreekumar et al. [63] (2020)^a^	Journal	India	LSG 50% OAGB 50%	43.5	48.8	NR	NR	IWQoL‐Lite (PD)	32 [32]^d^	18
Wei et al. [64] (2020)^a^	Journal	China	RYGB 4% LSG 96%	40.8	42.6	60	NR	IWQoL‐Lite (PD)	25 [25]	12 [1, 3, 6]
Carr et al. [21] (2022)^a^ ^,^ ^b^	Journal and analyses from author	Australia	ESG 26.2% LSG 73.8%	39.4	40.7	83.6	85.3 W 4.5 NR 3.1 A 1.6 O 1.5 B	IWQoL‐Lite (PD)	31 [61]	12 [6]
McGarrity et al. [22] (2022)^a^ ^,^ ^b^	Journal and analyses from author	United States	RYGB LSG	45.1	42.0	81	89 W	IWQoL‐Lite (PD)	148 [354]	18–36
Jassil et al. [65] (2023)^a^	Journal	United Kingdom	OAGB 16.3% RYGB 28.8% SG 54.9%	42.4	44.2	78.4	58.2 W 22.8 B 8.5 A 5.9 O 4.6 M	IWQoL‐Lite (PD)	125 [153]	12 [3, 6]
Mackenzie et al. [66] (2024)^a^	Journal	United Kingdom	LAGB 15% RYGB 38.8% SG 45.8%	42.6^c^	70.6		95.3 W 1.2 A 1.2 B 2.4 M	IWQoL‐Lite (PD)	83 [85]	36 [12]
By‐Band‐Sleeve Collective [19] (BBSCG; 2025)^a^	Journal	United Kingdom	RYGB 34% LAGB 34% SG 31%	46.4	47.3	76	85 W 7 B 3 A 2 M 2 O	IWQoL‐Lite (PD)	1160 [1346]	36 [1, 6, 12, 24]
Oseberg Trials										
Oseberg Trial (2025)^a^ ^,^ ^b^	Analyses from author	Norway	RYGB 50% SG 50%	SG 42.1 RYGB 42.4	SG 47.1 RYGB 48.2	66	95 W	IWQoL‐Lite (PD)	94 [354]	24 [12]
Svenavik et al. [23] (2023)^a^	Journal	Norway	RYGB 50% SG 50%	SG 42.1 RYGB 42.4	SG 47.1 RYGB 48.2	66	95 W	IWQoL‐Lite (PD)	109 [93]	36 [5 weeks, 12, 24]
Teen LABS										
Reiter‐Purtill et al. [67] (2020)^a^	Journal	United States	LAGB 2.2% SG 36.0% RYGB 61.9%	51.5	16.9	79.9	66.2 W	IWQoL‐Kids (SL)	124 [139]	12, 24 [6]
Xie [26] (2024)^b^	Dataset	United States	LAGB 5.8% SG 27.7% RYGB 66.5%	52.5	16.6	75.6	71.9 W 22.3 B 5.0 M 0.4 A 0.4 AI/AN	IWQoL‐Kids (SL)	218 [242]	12 [6, 24, 36, 48]

*Note:* Mean BMI, age (years), and female percentage (%) are baseline values unless otherwise specified.

Abbreviations: BMI, body mass index; NA, data/information not available; Surgery types: BPD/DS, biliopancreatic diversion with duodenal switch; ESG, endoscopic sleeve gastroplasty; LAGB, laparoscopic adjustable gastric banding; LSG, laparoscopic sleeve gastrectomy; OAGB, one anastomosis gastric bypass; RYGB, Roux‐en‐Y gastric bypass; SG, sleeve gastrectomy, VBG, vertical banded gastroplasty; IWQoL, Impact of Weight on Quality of Life scale; PD, Public Distress subscale; SL, Social Life subscale; A, Asian; AI/AN, American Indian/Alaska Native; B, Black; H, Hispanic; M, Mixed; O, Other; W, White.

^a^ Studies included in the pre–post analysis.

^b^ Studies included in the prediction of weight change.

^c^ Median value reported.

^d^ Baseline *n* reported by authors and upon which demographic statistics are based but may not reflect the number of participants in overall recruited sample.

### 3.2 Quality Assessment

A summary of the quality of the studies via the risk of bias tool can be found in see Table S2. Out of seven possible points, ratings ranged from 2 to 7 with a mean rating of 4.74 (SD = 1.14; median = 5).

### 3.3 Weight Stigma Outcomes

**FIGURE 2:**
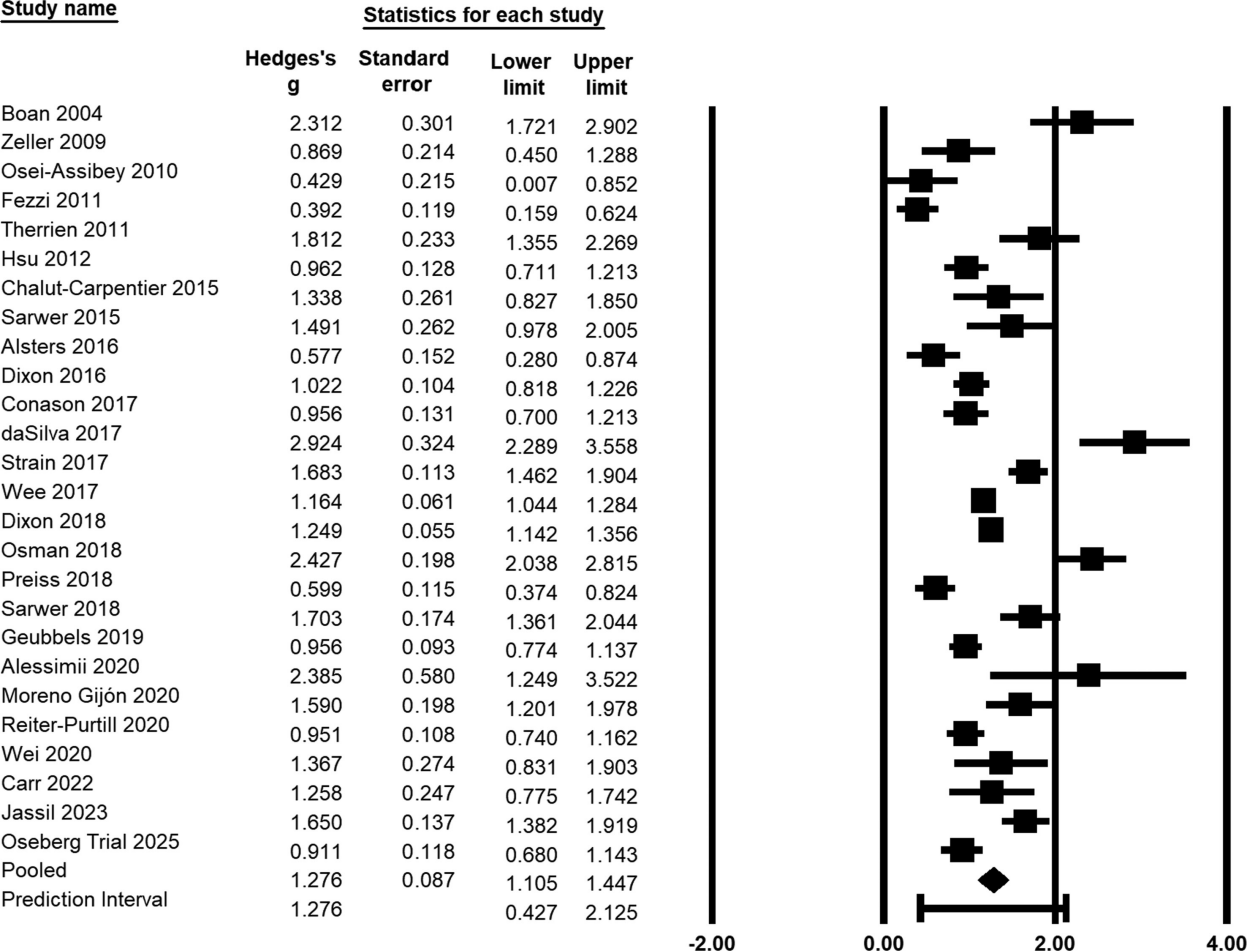
Forest plot of weight stigma change from pre‐surgery (T1) to 12 mos. post‐surgery (T2).

Analysis of 26 studies revealed a reduction of experienced weight stigma across the majority of studies with large effects observed for the T2 postoperative time point (see Figure 2; *g* = 1.28, CI: 1.11–1.45, *p* < 0.001, *Q* = 264.58, *p* < 0.001, *I* ^2^ = 91%). We conducted a sensitivity analysis excluding two studies that consisted of non‐adult aged samples [35, 67]. The effect size and heterogeneity remained statistically significant and nearly the same (*g* = 1.31, CI: 1.13–1.49). As a matter of comparison, we also conducted analyses on the additional subscales of the IWQoL‐Lite for this same postoperative time point (see Figures S1–S4; overall effects are reported in Table 2).

**TABLE 2:** Overall change effects over time for each subscale of the IWQoL‐Lite.

	*g*	CI	*Q*	*I* ^2^
Change T1 to T2				
Public distress	1.28	1.11–1.45*	264.58*	91%
Physical function	2.00	1.75–2.25*	340.64*	93%
Self‐esteem	1.33	1.15–1.50*	240.58*	90%
Sexual life	1.00	0.84–1.16*	221.51*	90%
Work	1.13	0.99–1.28*	157.62*	86%
Change T1 to T3				
Public distress	1.16	1.00–1.32*	155.85*	90%
Physical function	1.87	1.60–2.14*	229.32*	94%
Self‐esteem	1.26	1.08–1.44*	144.84*	91%
Sexual life	0.85	0.66–1.04*	150.95*	93%
Work	1.03	0.73–1.33*	429.48*	97%
Change T1 to T4				
Public distress	1.26	1.10–1.46*	52.35*	87%
Physical function	1.77	1.24–2.30*	170.01*	96%
Self‐esteem	1.36	1.17–1.56*	56.13*	88%
Sexual life	0.85	0.50–1.19*	229.70*	97%
Work	1.22	1.04–1.40*	23.45*	74%

* *p* < 0.001.

**FIGURE 3:**
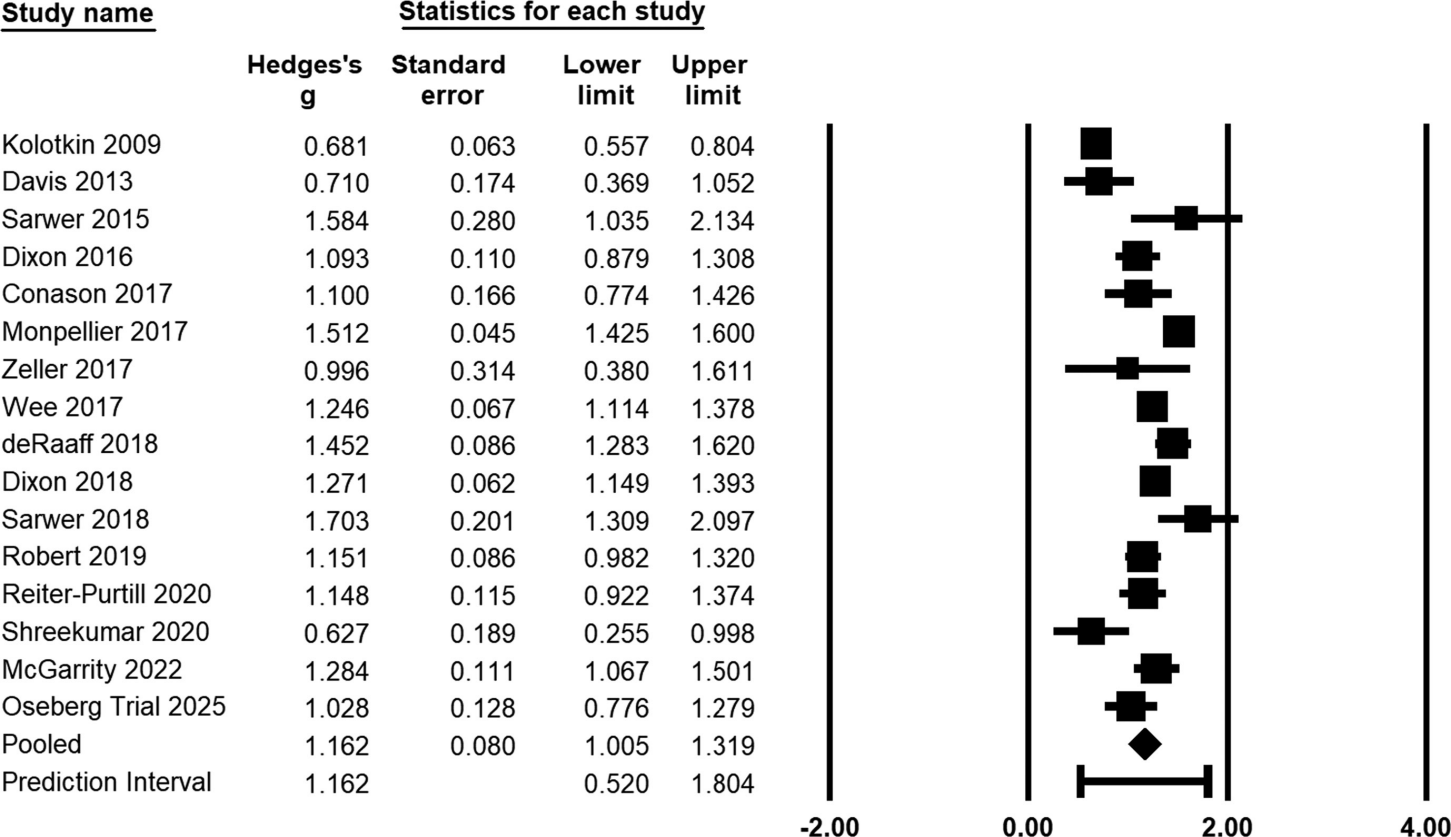
Forest plot of weight stigma change from pre‐surgery (T1) to 24 mos. post‐surgery (T3).

Analysis of 16 studies for the T3 postoperative time point also revealed a reduction of experienced weight stigma across all the studies with large effects observed (see Figure 3; *g* = 1.16, CI: 1.00–1.32, *p* < 0.001, *Q* = 155.85, *p* < 0.001, *I* ^*2*^ = 90%). We conducted a sensitivity analysis excluding two studies [53, 67] that consisted of non‐adult aged samples. The effect size and heterogeneity remained statistically significant and nearly the same (*g* = 1.17, CI: 1.00–1.34). As with the previous analysis, we conducted analyses on the additional subscales of the IWQoL‐Lite for this postoperative time point (see Figures S5–S8 and Table 2).

**FIGURE 4:**
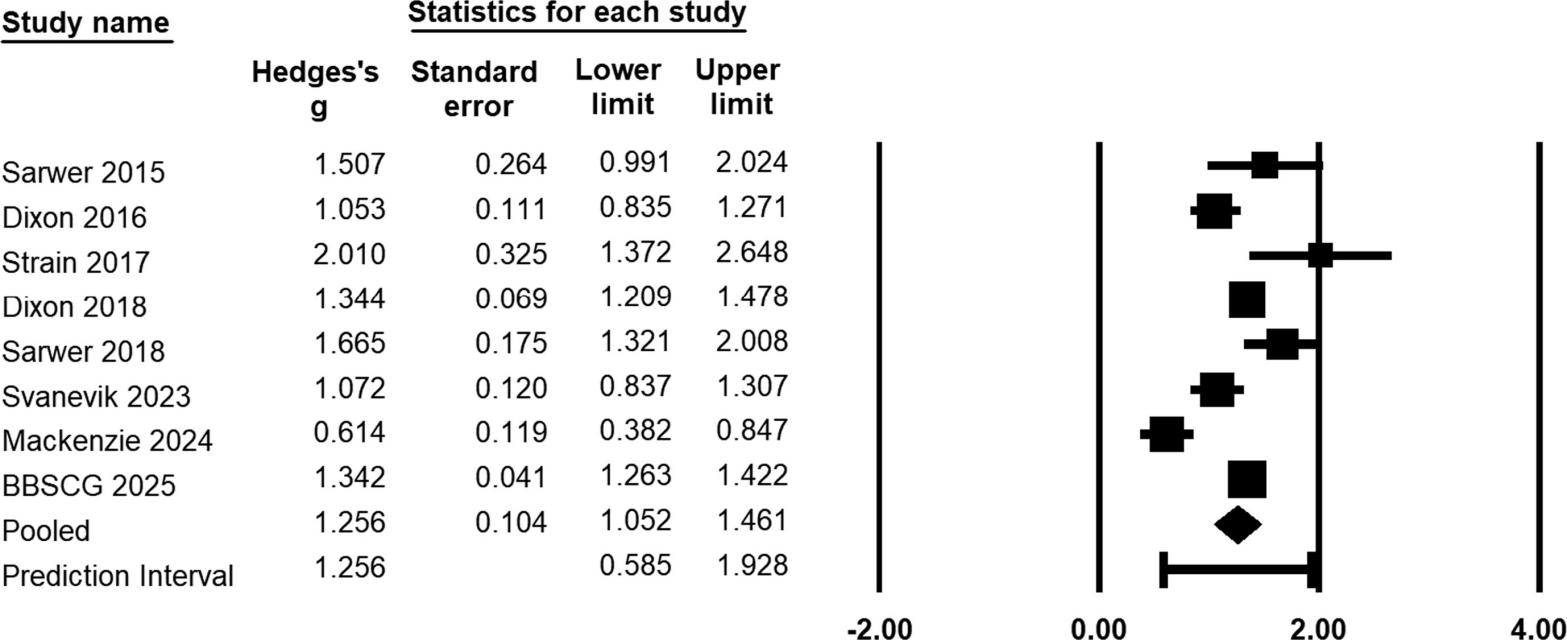
Forest plot of weight stigma change from pre‐surgery (T1) to 36 mos. post‐surgery (T4).

Analysis of eight studies for the T4 postoperative time point also revealed a decrease of experienced weight stigma across all of the studies with large effects observed (see Figure 4; *g* = 1.26, CI: 1.10–1.46, *p* < 0.001, *Q* = 52.35, *p* < 0.001, *I* ^2^ = 87%). The change in other subscales of the IWQoL‐Lite for this same postoperative time point was examined for comparison (see Figures S9–S12 and Table 2).

### 3.4 Moderators

We conducted our planned moderation analyses to explore potential sources of heterogeneity. The first moderator we assessed was surgery type, comparing Roux‐en‐Y Gastric Bypass (RYGB; *n* = 10) versus sleeve gastrectomy (SG)‐laparoscopic sleeve gastrectomy (LSG)–vertical sleeve gastrectomy (VSG) (*n* = 5) versus laparoscopic adjustable gastric banding (LAGB; *n* = 5) for the T2 postoperative time point. The effects for surgery types revealed that RYGB evidenced a more pronounced effect (*g* = 1.29, 95% CI: 1.02–1.57, *p* < 0.001) than SG‐LSG‐VSG (*g* = 1.16, 95% CI: 0.37–1.94, *p* = 0.004) and LAGB (*g* = 0.87, 95% CI: 0.60–1.14, *p* < 0.001). The overall difference between these three subgroups was not statistically significant (*p* = 0.098). However, when the comparison was limited to RYGB versus LAGB, the difference was statistically significant (*p* = 0.032), suggesting that although both types of surgeries have notable effects on experienced weight stigma, RYGB had a significantly larger effect. For the T3 postoperative time point, fewer studies (i.e., minimum of 5 per subgroup) were available for comparison than needed for conducting a reliable subgroup moderation analysis. That said, the effects for both surgery types on experienced stigma were large: RYGB (*n* = 8, *g* = 1.35, 95% CI: 1.04–1.65, *p* < 0.001) and LAGB (*n* = 4, *g* = 1.07, 95% CI: 0.88–1.27, *p* < 0.001). For the T4 postoperative time point, no moderation analysis was performed due to insufficient numbers of studies available to examine any of the different surgery types.

To determine the impact that study quality and gender of patients had on changes to experienced weight stigma pre‐to‐post MBS, meta‐regression analyses were performed. It appeared that the quality of the study was not associated with effect sizes for the T2 (*B* = −0.03, 95% CI = −0.18–0.12, *p* = 0.67), T3 (*B* = 0.04, 95% CI = −0.09–0.17, *p* = 0.58), and T4 outcomes (*B* = −0.24, 95% CI = −0.50–0.01, *p* = 0.06). Similarly, the gender of the study participant was not associated with effect sizes for the T2 (*B* = −0.002, 95% CI = −0.01–0.01, *p* = 0.65), T3 (*B* = −0.002, 95% CI = −0.01–0.01, *p* = 0.71), and T4 outcomes (*B* = −0.001, 95% CI = −0.02–0.01, *p* = 0.83).

### 3.5 Association With Postsurgical Weight Change

**FIGURE 5:**
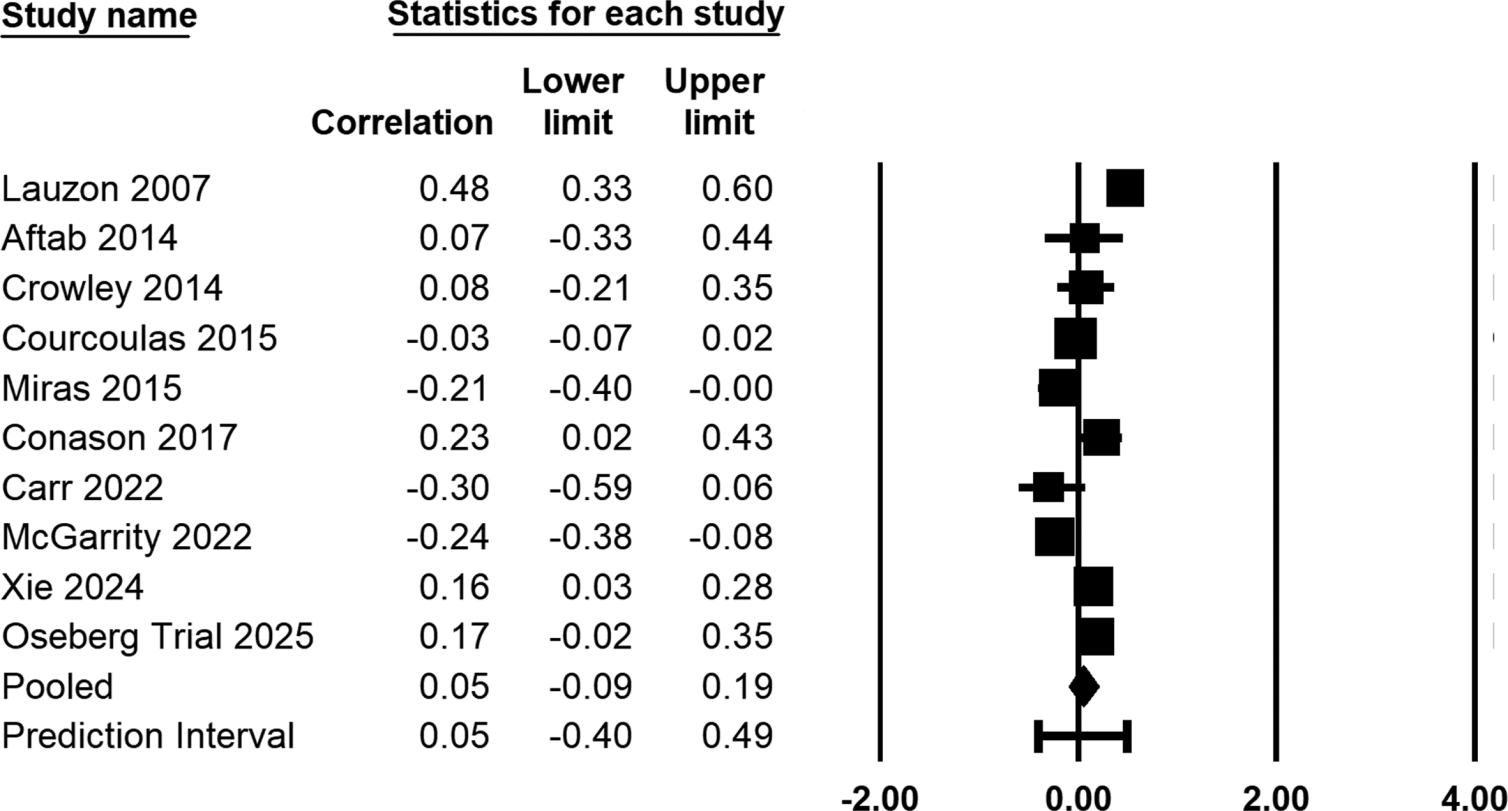
Forest plot of association between pre‐surgery stigma and weight change post‐surgery.

Ten studies were identified for assessing the association between presurgical experienced weight stigma and weight change following surgery. Analysis revealed a nonsignificant association of *r* = 0.05 (see Figure 5; CI: −0.09–0.19, *p* = 0.46, *Q* = 62.80, *p* < 0.001, *I* ^2^ = 86%). Moderators and other confounding variables in this relationship could not be examined as planned due to the small number of studies studying or reporting on these factors.

### 3.6 Publication Bias

For the T2 postoperative time point, Egger's regression intercept was 1.84, 95% CI: −0.86–4.53, *t* = 1.41, *p* = 0.09. Trim‐and‐fill analysis imputed three values below the mean, and the effect size was adjusted slightly downward from *g* = 1.28, CI: 1.11–1.45, to 1.16, CI: 0.98–1.34. For the T3 postoperative time point, Egger's regression intercept was −1.68, 95% CI: −5.36–2.01, *t* = 0.98, *p* = 0.17, suggesting no asymmetry or evidence of publication bias. Trim‐and‐fill analyses also did not impute any values above or below the mean. For the T4 postoperative time point, Egger's regression intercept was −0.47, 95% CI: −5.01–4.06, *t* = 0.26, *p* = 0.40, suggesting no asymmetry or evidence of publication bias. Trim‐and‐fill analysis imputed two values below the mean, and the effect size was adjusted slightly downward from *g* = 1.26, CI: 1.05–1.46, to 1.14, CI: 0.93–1.35.

## 4 Discussion

This meta‐analysis is the first to empirically synthesize a body of longitudinal studies examining experienced weight stigma and post‐MBS outcomes. Results of this meta‐analysis suggest that, as hypothesized, experienced weight stigma was reduced pre‐ to post‐MBS with large effect sizes. Moderator analyses indicated that RYGB had a larger effect than other surgery types at the T2 and T3 periods, which may be due to increased weight loss with RYGB as compared with the other procedures [68]. Indeed, the mean BMI loss following RYGB in the studies included in our meta‐analysis that reported BMI change (*M* = 15.36, SD = 4.45) was greater than that of other types of surgery (*M* = 10.49, SD = 3.75), and the magnitude of this effect was large (*d* = 1.19). The quality of study and gender of the study participants did not influence the findings. Contrary to original hypotheses, experienced weight stigma pre‐MBS was not associated with poorer weight outcomes.

Our findings that experienced weight stigma is reduced following MBS support a large body of work that indicates individuals in larger (vs. smaller) bodies experience more frequent weight‐based mistreatment [1, 2]. This pattern aligns with pervasive societal beliefs about the causes and controllability of weight. Specifically, laypersons and medical providers often overemphasize individual behaviors and underappreciate the biological, sociocultural, and environmental factors that impact body shape and weight [69]. Consequently, people appear more likely to assign negative characteristics (e.g., weak‐willed, lazy) to individuals with larger (vs. smaller) bodies [70]. Therefore, individuals who lose weight post‐MBS may be less vulnerable to weight‐based stereotyping. Notably, our findings that the additional subscales of the IWQoL‐Lite (Physical Functioning, Self‐esteem, Sexual Functioning, and Work) improve pre‐ to post‐MBS further support the several psychosocial benefits of undergoing MBS in addition to the physical health benefits.

Irrespective of potential reductions in weight stigma following MBS, qualitative research supports that past weight‐based maltreatment can be salient and detrimental to self‐esteem and emotional well‐being decades following stigmatizing events [71, 72]. Additional work is needed to capture how presurgical weight stigma may influence postsurgical mental health outcomes, such as self‐esteem, anxiety, and mood symptoms; notably, we only found three studies that reported on these relationships [22, 34, 57]. Internalized weight stigma, or self‐disparagement because of one's weight, is another potential consequence following experiences of weight‐based mistreatment. This review attempted to examine the impact of internalized weight stigma before and after MBS but was unable to do so because only two studies included this variable in longitudinal pre–post analyses [73, 74]. However, evidence from one systematic review conducted by Bidstrup and colleagues [75] suggests that internalized weight stigma may act as a mediator in the relationship between experiences of weight stigma and adverse health outcomes. Additionally, internalized weight stigma is associated with weight recurrence and disordered behavior [76], whereas reductions in weight self‐stigmatization are associated with greater weight loss [77] and eating self‐efficacy [78] in non‐MBS samples. Thus, future longitudinal work may consider examining how IWS relates to experienced weight stigma and post‐MBS outcomes.

Contrary to our hypothesis, we did not find a relationship between experienced weight stigma and weight change post‐MBS. This hypothesis was primarily driven by the associations between weight stigma and a variety of negative health outcomes found in previous research [79, 80], as well as negative psychosocial correlates that could contribute to lack of adherence (e.g., depression, anxiety) [81]. Supporting this speculation, a recent systematic review found associations between many of these psychosocial correlates of weight stigma to be predictive of reduced adherence following MBS [82]. Although our finding should be interpreted with caution given the limited number of studies (*n* = 10), there are some potential explanations for the minimal relationship we found between presurgical weight stigma and post‐MBS.

First, it may be that physiological changes following MBS are stronger determinants in weight loss following MBS than psychosocial factors [83]. Similarly, it could be that because weight loss substantially reduced experiences of weight stigma, it alleviated other factors (e.g., stress, depression) that contribute to reduced adherence. Indeed, another meta‐analysis found that depressive symptoms were greatly reduced from pre‐ to 12 months post‐MBS (*g* = 0.80) only somewhat less than the change we found for experienced weight stigma (*g* = 1.28) [84]. Other possibilities include the potential for the relationship between weight stigma and post‐MBS weight loss to be nonlinear or moderated by other variables. For example, when considering potential moderation of BMI, it is possible that individuals with a higher BMI at baseline are also more likely to endorse weight stigma and that these patients may lose more weight compared with those with a lower BMI at baseline. A meta‐analytic review indicates inconsistent findings regarding the relationship between pre‐MBS BMI and post‐MBS weight loss [85]. Overall, it appears that individuals with higher BMI experience greater absolute weight loss (measured in lbs./kg/BMI) but less excess weight loss (EWL), likely due to having more EWL to start with, so this hypothesis is not fully supported. Another example of a potential moderator is self‐efficacy levels. Meta‐analytic evidence indicates that self‐efficacy is a consistent predictor to postsurgical adherence [82], and it is possible that high self‐efficacy levels mitigate the behavioral impacts of experienced weight stigma on post‐MBS outcomes. Additional research into the relationship between presurgical weight stigma and post‐MBS weight outcomes may consider incorporating additional variables (e.g., BMI, social support, internalized weight stigma) as potential mediators or moderators to see if this relationship may be explained, in part, by indirect associations.

Another important contextual consideration of this study's findings is the postoperative timeframe of studies available for inclusion in this meta‐analysis (12‐, 24‐, and 36‐months post‐MBS). Most patients reach their nadir weight between 12 and 24 months post‐MBS [86, 87]. The “honeymoon period” of weight loss typically ends by 3 years, at which time some researchers have suggested “the real work begins” in terms of weight loss maintenance habits [88]. Clinically, patients commonly express frustration at differential treatment by others praising their weight loss and/or perpetuating ongoing stigma, especially in the context of weight recurrence after MBS [89]. As time goes on after surgery, the potential for life stressors to impact health behaviors is significant, with many patients reporting diminished social support following MBS [90]. Taken together, it is plausible that longer term follow‐up may yield different results for how experienced weight stigma pre‐MBS may affect the risk of suboptimal weight loss and weight recurrence over time.

Additionally, ongoing systemic barriers to accessing care and healthcare provider biases may place patients who have historically experienced high degrees of weight stigma at particular risk after MBS [79, 80]. It is promising that this meta‐analysis shows reductions in weight stigma post‐MBS; future research may highlight potential changes to healthcare utilization when patients experience weight recurrence post‐MBS. This may be a critical time when weight stigmatization from healthcare providers or perceptions of weight bias occur, resulting in reductions in important follow‐up care. Certainly, more research in this area is needed, yet the results presented here provide an important foundation for future studies.

The limited number of studies reporting on the relationships between experienced weight stigma and MBS outcomes by potential moderators (e.g., gender, surgery type) prevented us from exploring additional variables (e.g., preoperative weight, obesity‐related comorbidities, patient and provider surgery preferences) that may have impacted findings. Future research should examine these relationships in larger samples with more detailed characterization and standardized reporting practices to better clarify the interplay between weight stigma, gender, surgery type, and postoperative outcomes. Moreover, limited research prevented us from evaluating the impact of presurgical internalized weight bias on MBS outcomes. Despite these limitations, this study was the first review to include a robust meta‐analytic methodology to examine how presurgical weight stigma predicts post‐MBS outcomes. Specifically, this study was novel in synthesizing quantitative relationships between experienced weight stigma pre‐ to‐post MBS and weight loss up to 3 years after surgery. Moreover, this study analyzed the potential moderating impact of surgical type and gender on outcomes, as well as analyzed pre‐ and post‐MBS changes across IWQoL‐Lite subscales. Importantly, this review identified future research directions and clinical recommendations, highlighted in Table S3.

Weight stigma has long been identified as an important factor for obesity trajectories among non‐surgery populations. This meta‐analysis builds on the work of Bennett and colleagues [17] to summarize and explore using meta‐analytic techniques the longitudinal effects of experienced weight stigma pre‐ to post‐MBS and on post‐MBS weight. This study provides several promising areas of future research and ideas to enhance clinical practice among individuals who have had MBS and experience weight stigma. Specifically, results from this study revealed that weight stigma is reduced pre‐ to post‐MBS, other quality‐of‐life factors improve pre‐ to post‐MBS, and that greater weight stigma pre‐MBS is not directly associated with weight loss post‐MBS.

## 5 Conclusion

Weight stigma experiences are commonly reported in those with obesity, and patients pursuing MBS are at higher risk of discrimination, given higher categories of obesity. Past research suggests that weight stigmatization is related to several negative outcomes including depression, low self‐esteem, disordered eating, and metabolic changes impacting risk of weight gain [6, 16, 18]. Despite MBS being the most effective intervention for obesity, weight loss outcomes may be minimized due to impacts of pre‐surgical weight stigma. This study aimed to statistically analyze changes to experienced weight stigma before and after MBS, as well as the association between pre‐surgical weight stigma and postsurgical weight change. We found that experiences of weight stigma decrease following surgery at several timepoints (6–12 months, 13–24 months, 25–36 months), which is a benefit of undergoing MBS. Weight change post‐surgery was not associated with pre‐surgical experienced weight stigma. These findings should be interpreted with caution given the limited number of available studies. Additional longitudinal research on experienced weight stigma, postsurgical weight change, and other health outcomes in MBS samples is encouraged.

## Ethics Statement

This type of study is exempt from requiring ethical approval as there are no human subjects.

## Consent

This article does not contain any research with human participants performed by any of the authors.

## Conflicts of Interest

The authors declare no conflicts of interest.

## Supporting information


**Table S1:** Search strategies.
**Table S2:** Risk of bias by study and category.
**Table S3:** Future directions for research and clinical practice.
**Figure S1:** Forest plot of physical functioning change from pre‐surgery (T1) to 12 mos. post‐surgery (T2).
**Figure S2:** Forest plot of self‐esteem change from pre‐surgery (T1) to 12 mos. post‐surgery (T2).
**Figure S3:** Forest plot of sexual functioning change from pre‐surgery (T1) to 12 mos. post‐surgery (T2).
**Figure S4:** Forest plot of work functioning change from pre‐surgery (T1) to 12 mos. post‐surgery (T2).
**Figure S5:** Forest plot of physical functioning change from pre‐surgery (T1) to 24 mos. post‐surgery (T3).
**Figure S6:** Forest plot of self‐esteem change from pre‐surgery (T1) to 24 mos. post‐surgery (T3).
**Figure S7:** Forest plot of sexual functioning change from pre‐surgery (T1) to 24 mos. post‐surgery (T3).
**Figure S8:** Forest plot of work functioning change from pre‐surgery (T1) to 24 mos. post‐surgery (T3).
**Figure S9:** Forest plot of physical functioning change from pre‐surgery (T1) to 36 mos. post‐surgery (T4).
**Figure S10:** Forest plot of self‐esteem change from pre‐surgery (T1) to 36 mos. post‐surgery (T4).
**Figure S11:** Forest plot of sexual functioning change from pre‐surgery (T1) to 36 mos. post‐surgery (T4).
**Figure S12:** Forest plot of work functioning change from pre‐surgery (T1) to 36 mos. post‐surgery (T4).

## Data Availability

The data that support the findings of this study are available from the corresponding author upon reasonable request.
