# Supplementary material for: Experienced Weight Stigma Changes Following Bariatric Surgery: a Systematic Review and Meta‐Analysis
Source: Obes Rev. 2026 Jun 16;27(9):e70179. doi: 10.1111/obr.70179 (PMC13460558; doi:10.1111/obr.70179)
Supplement: Supplementary file 1 [file OBR-27-e70179-s001.docx]

**Experienced Weight Stigma Changes Following Bariatric Surgery: A Systematic Review and Meta-Analysis**

Hannah R. Farnsworth^1^, PsyD, Dakota L. Leget^2^, MA, Lara J. LaCaille^3^, PhD,

Rick A. LaCaille^3^, PhD, Afton M. Koball^4^, PhD, Alicia Niemann^3^,

Chloë Z. Backderf^3^, Carly R. Mastrian^3^, & Ellie M. Von Duyke^3^

^1^Department of Physical Medicine and Rehabilitation, University of Utah Health, 85 North Medical Drive, Salt Lake City, UT, 84132, USA

^2^Department of Clinical and Health Psychology, University of Florida, 1225 Center Drive, Gainesville, FL, 32601, USA

^3^Department of Psychology, University of Minnesota Duluth, 320 BohH 1207 Ordean Court, Duluth, MN, 55812, USA

^4^Department of Psychiatry and Psychology, Mayo Clinic, 200 First Street SW, Rochester, MN 55905, USA

**Author Note**

Hannah R. Farnsworth <https://orcid.org/0009-0004-2327-6373>

Dakota L. Leget <https://orcid.org/0000-0002-2538-6566>

Lara J. LaCaille <https://orcid.org/0000-0003-3503-3171>

Rick A. LaCaille <https://orcid.org/0000-0003-1783-3845>

Afton M. Koball <https://orcid.org/0000-0001-9145-3061>

Ellie M. Von Duyke <https://orcid.org/0009-0002-1704-8142>

Correspondence concerning this article should be addressed to Hannah Farnsworth, University of Utah Health, 85 North Medical Drive, Salt Lake City, UT, 84132, USA. Email: [hannah.farnsworth@hsc.utah.edu](mailto:hannah.farnsworth@hsc.utah.edu)

**Supplemental Table 1:** Search Strategies

**Search Strategy: General Search Terms**

| Database | Search Terms Used |
| --- | --- |
| CINAHL | (((MH "Weight Bias")) OR ((MH "Prejudice")) OR ((MH "Stigma")) OR ((MH "Implicit Bias")) OR ((MH "Discrimination")) OR ((MH "Microaggressions")) OR ((MH "Prejudice")) OR ((MH "Stereotyping")) OR ((MH "Shame")) OR (Stigma* OR (public AND distress) OR teas* OR bias* OR discriminat* OR prejudic* OR microaggress* OR stereotyp* OR harass* OR (anti-fat AND attitude) OR (weight AND self-stigma) OR (weight AND bias AND internaliz*) OR sham)) AND (((MH "Bariatric Surgery")) OR ((MH "Gastroplasty")) OR ((MH "Gastrectomy")) OR ((MH "Lipectomy")) OR ((MH "Anastomosis, Roux-en-Y")) OR ((MH "Gastric Bypass")) OR ((MH "Jejunoileal Bypass")) OR (Bariatric* OR (weight AND loss AND surgery) OR (obesity AND surg*) OR gastrectom* OR (metabolic AND surg*) OR gastroplast* OR lipectom* OR Roux-en-Y OR (biliopancreatic AND diversion) OR (scopinaro AND procedure*) OR (gastric AND banding) OR (lap AND band*) OR (gastric AND bypass) OR (duodenal AND switch) OR (gastric AND balloon*) OR (intragastric AND balloon*) OR (jejunoileal AND bypass) OR (gastric AND plication) OR (pre AND bariatric) OR (post AND bariatric))) |
| Google Scholar | We conducted individual title-only searches with each of the following stigma/shame terms with the following bariatric term. For example, the first combination of these terms would have looked like: “stigma” AND “bariatric.”  Stigma/shame key terms: “stigma”, “stigmatization”, “stigmatizing”, “stigmatisation”, “stigmatising”, “tease”, “teasing”, “discrimination”, “discriminating”, “prejudice”, “microaggression”, “shame”, “shaming”, “harassment”, “harassing”, “distress”, “bias”, “biased”, “anti-fat attitudes”, “stereotype”, “stereotyping”  Bariatric key terms: “bariatric”, “metabolic surgery”, “weight loss surgery”, “obesity surgery”, “gastrectomy”, “gastroplasty”, “lipectomy”, “gastric bypass”, “gastric band”, “gastric banding”, “lap band”, “gastric balloon”, “biliopancreatic diversion”, “scopinaro procedure”, “duodenal switch”, “jejunoileal bypass” |
| ProQuest Dissertations and Theses Global | (TITLE,ABSTRACT,IF,SUBJECT((weight NEAR/5 self-stigma) OR weight bias internaliz* OR internaliz* weight bias OR body shame OR (body NEAR/5 shame) OR shame)) AND (TITLE,ABSTRACT,IF,SUBJECT(bariatric* OR bariatric surger* OR weight loss surg* OR obesity surg* OR gastrectomy* OR WLS OR post-bariatric OR metabolic surgery OR gastroplasty* OR lipectomy* OR pre-bariatric OR roux-en-y gastric bypass* OR RYGB OR RNY OR biliopancreatic diver* OR biolo-pancreatic diver* OR BPD OR scopinaro procedure* OR sleeve gastrect* OR SG OR VSG OR vertical banded gastroplasty OR VBG OR laparoscopic sleeve gastrect* OR LSG OR laparoscopic adjustable gastric band* OR LAGB OR lap band* OR gastric bypass* OR OAGB OR GB OR GBP OR duodenal switch OR DS OR gastric balloon* OR intragastric balloon* OR IGB OR gastric band* OR adjustable gastric band* OR A band* OR jujunoileal bypass* OR JIB OR gastric plication* OR LGCP)) |
| PsycInfo | ((DE "Stigma" OR DE "Mental Health Stigma" OR DE "Self-Stigma") OR (DE "Teasing") OR (DE "Implicit Bias" OR DE "Prejudice") OR (DE "Discrimination") OR (DE "Microaggression") OR (DE "Shame") OR (DE "Stereotyped Attitudes") OR (Stigma* OR (public AND distress) OR teas* OR bias* OR discriminat* OR prejudic* OR microaggress* OR stereotyp* OR harass* OR (anti-fat AND attitude) OR (weight AND self-stigma) OR (weight AND bias AND internaliz*) OR sham*)) AND ((DE "Bariatric Surgery") OR (Bariatric* OR (weight AND loss AND surgery) OR (obesity AND surg*) OR gastrectom* OR (metabolic AND surg*) OR gastroplast* OR lipectom* OR Roux-en-Y OR (biliopancreatic AND diversion) OR (scopinaro AND procedure*) OR (gastric AND banding) OR (lap AND band*) OR (gastric AND bypass) OR (duodenal AND switch) OR (gastric AND balloon*) OR (intragastric AND balloon*) OR (jejunoileal AND bypass) OR (gastric AND plication) OR (pre AND bariatric) OR (post AND bariatric))) |
| PubMed | (((((((((“Bariatric Surgery” [Mesh]) OR (“Gastrectomy” [Mesh])) OR (“Gastroplasty” [Mesh])) OR (“Anastomosis, Roux-en-Y” [Mesh])) OR (“Biliopancreatic Diversion” [Mesh])) OR (“Gastric Bypass” [Mesh])) OR (“Gastric Balloon” [Mesh])) OR (“Jejunoileal Bypass” [Mesh])) OR (Bariatric[tiab] OR weight loss surgery[tiab] OR obesity surg*[tiab] OR gastrectom*[tiab] OR metabolic surg*[tiab] OR gastroplast*[tiab] OR lipectom*[tiab] OR Roux-en-Y[tiab] OR biliopancreatic diversion[tiab] OR scopinaro procedure*[tiab] OR gastric banding[tiab] OR lap band*[tiab] OR gastric bypass[tiab] OR duodenal switch[tiab] OR gastric balloon*[tiab] OR intragastric balloon*[tiab] or jejunoileal bypass[tiab] OR gastric plication[tiab] OR pre bariatric[tiab] OR post bariatric[tiab])) AND ((((((“Weight Prejudice” [Mesh]) OR (“Perceived Discrimination” [Mesh])) OR (“Prejudice” [Mesh])) OR (“Microaggression” [Mesh])) OR (“Stereotyping” [Mesh])) OR (Stigma*[tiab] OR public distress[tiab] OR teas*[tiab] OR bias*[tiab] OR discriminat*[tiab] OR prejudic*[tiab] OR microaggress*[tiab] OR stereotyp*[tiab] OR harass*[tiab] OR anti fat attitudes[tiab] OR weight self-stigma[tiab] OR weight bias internaliz*[tiab] OR sham*[tiab])) |
| Web of Science Core Collection | TS=(bariatric* OR "bariatric surgery" OR bariatric surger* OR "weight loss surg*" OR obesity surg* OR "obes* surg*" OR gastrectomy* OR WLS OR post-bariatric OR "metabolic surgery" OR gastroplasty* OR lipectomy* OR pre-bariatric) AND TS=("roux-en-y gastric bypass*" OR RYGB OR RNY OR "biliopancreatic diver*" OR "biolo-pancreatic diver*" OR BPD OR "scopinaro procedure*" OR "sleeve gastrect*" OR SG OR VSG OR "vertical banded gastroplasty" OR VBG OR "laparoscopic sleeve gastrect*" OR LSG OR "laparoscopic adjustable gastric band*" OR LAGB OR "lap band*" OR "gastric bypass*" OR OAGB OR GB OR GBP OR "duodenal switch" OR DS OR "gastric balloon*" OR "intragastric balloon*" OR IGB OR "gastric band*" OR "adjustable gastric band*" OR "A band*" OR "jujunoileal bypass*" OR JIB OR "gastric plication*" OR LGCP) AND TS=("weight stigma*" OR stigma* OR public distress OR teas* OR "weight bias" OR (weight NEAR/5 bias*) OR discriminat* OR prejudice OR microaggression OR "fat shaming" OR "anti-fat attitude" OR stereotype OR harassment) AND TS=(weight NEAR/5 self-stigma OR "weight bias internaliz*" OR "internaliz* weight bias" OR "body shame" OR (body NEAR/5 shame) OR shame) |

**Search Strategy: Measure-Specific Search**

| Database | Search Terms Used |
| --- | --- |
| ProQuest Dissertations & Theses Global | We conducted individual full-text searches with each of the following stigma/shame key measures with the “bariatric” key term. For example, the first combination of these terms would have looked like: “Objectified Body Consciousness Scale” AND “bariatric.”  Stigma/shame measures: "Objectified Body Consciousness Scale", "Weight Bias Internalization Scale", "Weight Self Stigma Questionnaire", "Acceptance and Action Questionnaire for Weight Related Difficulties", "Weight- and Body-Related Shame and Guilt Scale", "Weight Based Rejection Sensitivity", "Weight-Focused Forms of Self-Criticising/Attacking and Self-Reassuring Scale", "Weight Related Abuse Questionnaire", "Obesity and Weight-Loss Quality-of-Life Questionnaire", "Feelings and Thoughts About Weight Scale", "Experience of Weight Based Discrimination", "Perceived Weight Discrimination", "Perceived Weight-Based Stigmatization Scale", "Perception of Teasing Scale", "Physical Appearance Related Teasing Scale", "Quality of Life for Obesity Surgery Questionnaire", "Stigma Preferences Scale", “Impact of Weight on Quality of Life”, "Weight Loss Surgery and Work Expectations Questionnaire", “Index of Weight Related Stress Scale”, “Everyday Discrimination Scale”, “Obesity Psychosocial State Questionnaire” |
| Google Scholar | We conducted individual full-text searches with each of the following stigma/shame key measures with the “bariatric” key term. For example, the first combination of these terms would have looked like: “Objectified Body Consciousness Scale” AND bariatric  Stigma/shame measures: "Objectified Body Consciousness Scale", "Weight Bias Internalization Scale", "Weight Self Stigma Questionnaire", "Acceptance and Action Questionnaire for Weight Related Difficulties", "Weight- and Body-Related Shame and Guilt Scale", "Weight Based Rejection Sensitivity", "Weight-Focused Forms of Self-Criticising/Attacking and Self-Reassuring Scale", "Weight Related Abuse Questionnaire", "Obesity and Weight-Loss Quality-of-Life Questionnaire", "Feelings and Thoughts About Weight Scale", "Experience of Weight Based Discrimination", "Perceived Weight Discrimination", "Perceived Weight-Based Stigmatization Scale", "Perception of Teasing Scale", "Physical Appearance Related Teasing Scale", "Quality of Life for Obesity Surgery Questionnaire", "Stigma Preferences Scale", “Impact of Weight on Quality of Life”, "Weight Loss Surgery and Work Expectations Questionnaire", “Index of Weight Related Stress Scale”, “Everyday Discrimination Scale”, “Obesity Psychosocial State Questionnaire” |

**Supplemental Table 2.** Risk of Bias by Study and Category

|  | Eligibility & selection criteria | Participant representativeness | Enrolled all eligible participants | Sufficient sample size of ≥ 47 | Intervention delivered consistently | Measures valid/reliable & assessed consistently across all participants | Follow-up loss < 20% | Total score |
| --- | --- | --- | --- | --- | --- | --- | --- | --- |
| Boan et al. (2004)^a^ | - | + | + | - | + | + | + | 5 |
| Lauzon (2007)^d^ | + | + | + | - | - | + | - | 4 |
| Kolotkin et al. (2009)^b^ | + | + | + | + | - | + | - | 5 |
| Zeller et al. (2009)^a^ | + | - | - | - | + | + | + | 4 |
| Osei-Assibey et al. (2010)^a^ | + | + | + | - | - | + | + | 5 |
| Fezzi et al. (2011)^a^ | + | + | + | + | + | + | + | 7 |
| Therrien et al. (2011)^a^ | + | + | + | + | - | + | - | 5 |
| Hsu (2012)^a^ | + | + | + | + | - | + | - | 5 |
| Davis (2013)^b^ | + | + | - | - | + | + | - | 4 |
| Aftab et al. (2014)^d^ | + | + | + | - | - | + | - | 4 |
| Crowley (2014)^d^ | + | + | - | - | + | + | - | 4 |
| Chalut-Carpentier et al. (2015)^a^ | - | + | + | - | - | + | - | 3 |
| Courcoulas et al. (2015)^d^ | + | + | ? | + | - | + | + | 5 |
| Miras et al. (2015)^d^ | + | + | - | + | - | + | + | 5 |
| Sarwer et al. (2015)^a,b,c^ | + | - | + | - | + | + | + | 5 |
| Alsters (2016)^a^ | + | + | - | + | + | + | - | 5 |
| Dixon et al. (2016)^a,b,c^ | + | + | + | + | + | + | + | 7 |
| Conason et al. (2017)^a,b,d^ | - | + | - | + | - | + | - | 3 |
| da Silva et al. (2017)^a^ | + | - | + | + | - | + | + | 5 |
| Monpellier et al. (2017)^b^ | + | + | - | + | - | + | - | 4 |
| Strain et al. (2017)^a,c^ | + | + | - | - | + | + | - | 4 |
| Wee et al. (2017)^a,b^ | + | + | - | + | - | + | +^a^ /-^b^ | 5^a^ /4^b^ |
| Zeller et al. (2017)^b^ | + | - | + | - | - | + | + | 4 |
| de Raaff et al. (2018)^b^ | + | - | + | + | - | + | - | 4 |
| Dixon et al. (2018)^a,b,c^ | + | + | + | + | + | + | +^a^ /-^bc^ | 7^a^ /6^bc^ |
| Osman et al. (2018)^a^ | + | + | + | + | - | + | + | 6 |
| Preiss et al. (2018)^a^ | + | + | + | + | - | - | + | 5 |
| Sarwer et al. (2018)^a,b,c^ | + | - | + | + | - | + | + | 5 |
| Geubbels et al. (2019)^a^ | + | + | - | + | + | + | + | 6 |
| Robert et al. (2019)^b^ | + | + | + | + | + | + | - | 6 |
| Alessimii (2020)^a^ | + | + | - | - | + | + | - | 4 |
| Moreno Gijón et al. (2020)^a^ | + | + | + | + | - | + | + | 6 |
| Reiter-Purtill et al. (2020)^a,b^ | + | - | - | + | - | + | + | 4 |
| Shreekumar et al. (2020)^b^ | - | - | - | - | - | + | + | 2 |
| Wei (2020)^a^ | - | + | ? | - | - | + | + | 3 |
| Carr et al. (2022)^a,d^ | + | - | - | - | + | + | - | 3 |
| McGarrity et al. (2022)^b,d^ | - | + | + | + | - | + | - | 4 |
| Jassil et al. (2023)^a^ | + | + | ? | + | + | + | + | 6 |
| Mackenzie et al. (2024)^c^ | + | + | - | + | + | + | + | 6 |
| Xie et al. (2024)^d^ | + | - | - | + | - | + | + | 4 |
| By-Band-Sleeve Collective (2025)^c^ | + | + | - | + | + | + | + | 6 |
| Oseberg Trial (2025)^a,b,c,d^ | + | + | - | + | + | + | + | 6 |
| *Note.* + criterion met, - criterion not met, ? unspecified  ^a^denotes studies included for pre-post 0-12 months, ^b^denotes studies included for pre-post 12-24 months, ^c^denotes studies included for pre-post 24-36 months, ^d^denotes studies included for prediction of weight change | | | | | | | | |

**Supplemental Table 3.** Future Directions for Research and Clinical Practice

Future Research Directions

1. Evaluate long-term (>36 mo) follow-up of EWS and IWS measures and surgical outcomes.

2. Report EWS and IWS findings with relationships of these on surgical outcomes.

3. Report data on potential moderators of these relationships (e.g., gender, racial/ethnic, age).

4. Explore how EWS and IWS evolve post-MBS and whether this impacts post-surgical outcomes.

5. Consider qualitative investigations of the relationship between EWS and IWS pre- and post-MBS and impacts on life after surgery.

Future Clinical Directions

1. Assess weight stigma pre- and post-MBS.
   a. Suggested measures for EWS: IWQoL-Lite,^30^ Quality of Life for Obesity Surgery (QOLOS),^92^ and Stigmatizing Situations Inventory (SSI)^93^
   b. Suggested measures for IWS: Weight Bias Internalization Scale (WBIS),^94^ Weight Self-Stigma Questionnaire (WSSQ),^95^ Acceptance and Action Questionnaire for Weight (AAQ-W)^96^

2. Query patient’s experiences of weight stigma during clinical interactions.

3. Provide education on the deleterious impacts of weight stigma on health, quality of life, well-being, and risk of morbidity and mortality.

4. Develop a standardized follow up plan with patients post-MBS to provide ongoing support for stigmatizing experiences after MBS.

5. Be an advocate! Consider donating time or monetary resources to organizations that promote awareness and engage in activism of weight stigma such as the Obesity Action Coalition.^97^

**Supplemental Figure 1.** Forest Plot of Physical Functioning Change from Pre-surgery (T1) to 12 mos. Post-surgery (T2)

**Supplemental Figure 2.** Forest Plot of Self-esteem Change from Pre-surgery (T1) to 12 mos. Post-surgery (T2)

**Supplemental Figure 3.** Forest Plot of Sexual Functioning Change from Pre-surgery (T1) to 12 mos. Post-surgery (T2)

**Supplemental Figure 4.** Forest Plot of Work Functioning Change from Pre-surgery (T1) to 12 mos. Post-surgery (T2)

**Supplemental Figure 5.** Forest Plot of Physical Functioning Change from Pre-surgery (T1) to 24 mos. Post-surgery (T3)

**Supplemental Figure 6.** Forest Plot of Self-esteem Change from Pre-surgery (T1) to 24 mos. Post-surgery (T3)

**Supplemental Figure 7.** Forest Plot of Sexual Functioning Change from Pre-surgery (T1) to 24 mos. Post-surgery (T3)

**Supplemental Figure 8.** Forest Plot of Work Functioning Change from Pre-surgery (T1) to 24 mos. Post-surgery (T3)

**Supplemental Figure 9.** Forest Plot of Physical Functioning Change from Pre-surgery (T1) to 36 mos. Post-surgery (T4)

**Supplemental Figure 10.** Forest Plot of Self-esteem Change from Pre-surgery (T1) to 36 mos. Post-surgery (T4)

**Supplemental Figure 11.** Forest Plot of Sexual Functioning Change from Pre-surgery (T1) to 36 mos. Post-surgery (T4)

**Supplemental Figure 12.** Forest Plot of Work Functioning Change from Pre-surgery (T1) to 36 mos. Post-surgery (T4)
